# Eligibility Criteria for Different Platinum-Based Chemotherapy Regimens in Metastatic Urothelial Carcinoma

**DOI:** 10.7759/cureus.66520

**Published:** 2024-08-09

**Authors:** Faisal Azam, Hulayel Alharbi, Abdulraheem Alshangiti, Abdul Rehman Zar Gul, Nedal Bukhari, Mohamed Ouda, Syed Anwar Hussain, Fahad Ibnshamsah

**Affiliations:** 1 Department of Adult Medical Oncology, King Fahad Specialist Hospital, Dammam, SAU; 2 Department of Medical Oncology, National Cancer Center of Care and Research, Doha, QAT; 3 Department of Medical Oncology, Prince Sultan Military Medical City, Riyadh, SAU; 4 Department of Medical Affairs, Merck Limited Saudi Arabia, an Affiliate of Merck KGaA, Riyadh, SAU; 5 Department of Oncology and Metabolism, Medical School, University of Sheffield, Sheffield, GBR

**Keywords:** metastatic urothelial carcinoma, hematological parameters, eligibility criteria, cisplatin-based chemotherapy, carboplatin-based therapy

## Abstract

Introduction

Bladder cancer is one of the most prevalent cancers worldwide, with significant morbidity and mortality rates. Treatment options for metastatic urothelial carcinoma (mUC) primarily include platinum-based chemotherapy. Cisplatin-based chemotherapy is conventionally used for treating mUC, but many patients are ineligible due to various factors such as poor performance status, creatinine clearance, neuropathy, and cardiac function. Carboplatin-based therapy is another alternative, which typically yields less favorable outcomes. Some centers use split-dose cisplatin for treating patients with comorbidities and impaired renal function, broadening cisplatin’s spectrum. While eligibility criteria for full-dose cisplatin are well-established, those for split-dose cisplatin and carboplatin lack strong evidence. This study aims to assess the recommended criteria for full-dose cisplatin, split-dose cisplatin, and carboplatin regimens in real-world settings, including hematological parameters for patients with mUC.

Methods

A cross-sectional web-based survey was conducted among 136 oncologists from 21 countries, assessing criteria such as creatinine clearance, Eastern Cooperative Oncology Group (ECOG) performance status (PS), neurotoxicity, hearing loss, heart failure classification, and hematological parameters.

Results

The survey revealed diverse preferences among 113 oncologists treating mUC, regarding the eligibility criteria for each chemotherapy regimen with 81% prioritizing full-dose cisplatin, 21% split-dose cisplatin, and 14% carboplatin regimens. Criteria for all three regimens included specific thresholds. For full-dose cisplatin, the preferred criteria included creatinine clearance ≥60 mL/min, ECOG PS ≤1, grade 1 neuropathy, grade 1 deafness, New York Heart Association (NYHA) heart failure ≤class II with ≥50% cardiac ejection fraction, and normal blood parameters. Split-dose cisplatin criteria were creatinine clearance ≥40 mL/min, ECOG PS ≤2, grade 1 neuropathy, grade 1 deafness, NYHA heart failure ≤class II with ≥50% cardiac ejection fraction, and normal blood parameters. Carboplatin eligibility criteria were creatinine clearance ≥30, ECOG PS ≤2, grade ≤2 neuropathy, grade ≤2 deafness, NYHA heart failure ≤class II with ≥50% cardiac ejection fraction, and normal blood parameters. Hematological parameters were deemed crucial for all regimens, particularly stringent for carboplatin-based chemotherapy.

Conclusion

The study underscores the importance of renal function and hematological parameters in determining chemotherapy eligibility for patients with mUC. It highlights the importance of precise treatment criteria in mUC management, with hematological factors playing a significant role. Standardized criteria and further research are warranted to optimize treatment outcomes and minimize adverse events associated with chemotherapy regimens. Understanding the preferences of oncologists globally can facilitate tailored treatment approaches and improve patient care in the management of mUC.

## Introduction

Bladder cancer ranks as the 10th most prevalent cancer worldwide and holds the sixth highest number of estimated cases among all cancers [1,2]. It is the seventh most common cancer among men and ranks 17th among women [3]. Bladder cancer can be categorized as urothelial or non-urothelial, including squamous cell carcinoma, adenocarcinoma, small cell carcinoma, or sarcoma [4]. Urothelial carcinoma, originating from the urothelial cells, encompasses cancers of the urethra, bladder, ureter, and renal pelvis [5]. Approximately 5-10% of urothelial carcinomas arise in the upper urinary tract (renal pelvis and ureter), while 90-95% occur in the lower tract (bladder and urethra) [1]. Despite its prevalence, bladder cancer research progress has been limited over the past three decades.

Real-world studies indicate that 80-90% of patients with metastatic urothelial carcinoma (mUC) receive full-dose cisplatin chemotherapy as the conventional first-line treatment [1]. A prospective phase II clinical trial (NCT01490437), examining the combination of pemetrexed and cisplatin in 42 patients with mUC having Eastern Cooperative Oncology Group Performance Status (ECOG PS) 0-2, highlighted this regimen to be well-tolerated as a first-line treatment [6]. In another phase II clinical trial (NCT01524991) with 36 chemotherapy-naive patients having mUC, the combination of gemcitabine-cisplatin plus ipilimumab demonstrated a 1-year overall survival rate of 61%, and a 69% objective response rate, with Grade ≥3 adverse events in 81% of patients, primarily hematologic [7]. However, many patients are deemed ineligible based on various criteria like performance status, creatinine clearance, neuropathy, and cardiac function.

In several regions of the world, split-dose cisplatin is administered to full-dose cisplatin-ineligible patients with mUC to reduce renal toxicity [8,9]. A phase II clinical trial (NCT04602078) explored the combination of atezolizumab, split-dose cisplatin, and gemcitabine in 66 patients with histologically confirmed mUC, suggesting potentially favorable outcomes compared to other combinations [10]. Notably, split-dose cisplatin has also been employed in treating lung cancer [11]. Alternatively, using carboplatin-based chemotherapy in patients ineligible for cisplatin yielded comparatively poor outcomes. In a randomized phase II study (EORTC 30986), gemcitabine-cisplatin was compared with gemcitabine-carboplatin in 238 chemotherapy-naïve patients with mUC having ECOG PS 2 and poor renal performance, revealing a comparably acceptable toxicity profile for gemcitabine-carboplatin [12].

Decision-making in selecting treatment options for mUC relies on the Galsky and Bajorin risk factor criteria, but it deems patients ineligible for platinum-based chemotherapy [13]. A similar scenario was observed in a survey-based study, where the criteria for patients ineligible for platinum-based chemotherapy were highlighted [14]. Moreover, existing studies failed to address the hematological considerations necessary for holding patients eligible for platinum-based chemotherapy. The study aims to assess criteria, including hematological parameters, for initial treatment eligibility of mUC patients with full-dose cisplatin, split-dose cisplatin, and carboplatin-based chemotherapy. This includes gathering global uro-oncologists' opinions in a real-world context.

## Materials and methods

Study design and participants

A cross-sectional survey was conducted using an online questionnaire created in Microsoft Office Forms. The participants comprised practicing oncologists from various countries. The questionnaire was disseminated to approximately 1200 oncologists, via email for four months, from August to November 2023. The inclusion criteria mandated the active involvement of oncologists in treating patients with mUC. Oncologists not treating patients with mUC were excluded from the survey.

A team of experts from Saudi Arabia, specializing in treating patients with mUC, provided valuable inputs to originally develop the questionnaire (included in Table 1). It consisted of an initial consent section followed by four parts. The first part comprised eight questions related to basic demographics. Following that, the second, third, and fourth parts contained six questions each, assessing experiences regarding full-dose cisplatin eligibility, split-dose cisplatin usage, and carboplatin-based chemotherapy eligibility, respectively. The survey typically took about five minutes to complete.

**Table 1:** Survey questionnaire

(A) Personal Details
1. Name:
2. Email address:
3. Specialization:
4. Hospital associated with:
5. Hospital sector: Private/ Public/ University
6. Country where you practice:
7. Years of experience: (number)
8. Number of patients with bladder cancer you treat per year:
A. <10
B. 10–30
C. More than 30
(B) Survey Questions
For patients with advanced urothelial bladder cancer (UC), what would you consider for 1st line of treatment?
A. Full-dose cisplatin-based chemotherapy
B. Split-dose cisplatin-based chemotherapy
C. Carboplatin-based chemotherapy
D. Some other options, please specify
I. Full-Dose Cisplatin Eligibility
1. You would consider a patient with advanced UC fit for full-dose cisplatin if the creatinine clearance is
A. ≥20 mL/min
B. ≥30 mL/min
C. ≥40 mL/min
D. ≥60 mL/min
E. Not considered
F. Some other options, please specify
2. You would consider a patient with advanced UC fit for full-dose cisplatin if the ECOG PS is
A. 0
B. ≤1
C. ≤2
D. ≤3
E. Not considered
F. Some other options, please specify
3. You would consider a patient with advanced UC fit for full-dose cisplatin if the neuropathy is
A. ≤Grade 1
B. ≤Grade 2
C. ≤Grade 3
D. Not considered
E. Some other options, please specify
4. You would consider a patient with advanced UC fit for full-dose cisplatin if the hearing loss is
A. ≤Grade 1
B. ≤Grade 2
C. ≤Grade 3
D. Not considered
E. Some other options, please specify
5. You would consider a patient with advanced UC fit for full-dose cisplatin if the cardiac status is
i. NYHA Heart Failure
A. ≤Class II
B. ≤Class III
C. ≤Class IV
D. Not considered
E. Some other options, please specify
ii. Cardiac Ejection Fraction
A. <40%
B. 40%–50%
C. ≥50%
D. Not considered
E. Some other options, please specify
6. You would consider a patient with advanced UC fit for full-dose cisplatin if the hematological criteria are
A. Hemoglobin ≥11.0 g/dL (considered – not considered)
B. Platelet count ≥100,000 × 10^9^/L or 100,000/mm^3^ (considered – not considered)
C. Neutrophil count ≥1500 × 10^9^/L or 1500/mm^3^ (considered – not considered)
D. Some other options, please specify
II. Split-Dose Cisplatin Usage
1. You would consider a patient with advanced UC fit for split-dose cisplatin if the creatinine clearance is
A. ≥20 mL/min
B. ≥30 mL/min
C. ≥40 mL/min
D. ≥60 mL/min
E. Not considered
F. Some other options, please specify
2. You would consider a patient with advanced UC fit for split-dose cisplatin if the ECOG PS is
A. 0
B. ≤1
C. ≤2
D. ≤3
E. Not considered
F. Some other options, please specify
3. You would consider a patient with advanced UC fit for split-dose cisplatin if the neuropathy is
A. ≤Grade 1
B. ≤Grade 2
C. ≤Grade 3
D. Not considered
E. Some other options, please specify
4. You would consider a patient with advanced UC fit for split-dose cisplatin if the hearing loss is
A. ≤Grade 1
B. ≤Grade 2
C. ≤Grade 3
D. Not considered
E. Some other options, please specify
5. You would consider a patient with advanced UC fit for split-dose cisplatin if the cardiac status is
i. NYHA Heart Failure
A. ≤Class II
B. ≤Class III
C. ≤Class IV
D. Not considered
E. Some other options, please specify
ii. Cardiac Ejection Fraction
A. <40%
B. 40%–50%
C. ≥50%
D. Not considered
E. Some other options, please specify
6. You would consider a patient with advanced UC fit for split-dose cisplatin if the hematological criteria are
A. Hemoglobin ≥11.0 g/dL (considered – not considered)
B. Platelet count ≥100,000´10^9^/L or 100,000/mm^3 ^(considered – not considered)
C. Neutrophil count ≥1500´10^9^/L or 1500/mm^3^ (considered – not considered)
D. Some other options, please specify
III. Carboplatin Eligibility
1. You would consider a patient with advanced UC fit for carboplatin if the creatinine clearance is
A. ≥20 mL/min
B. 20–30 mL/min
C. 30–40 mL/min
D. ≥40 mL/min
E. Not considered
F. Some other options, please specify
2. You would consider a patient with advanced UC fit for carboplatin if the ECOG PS is
A. 0–2
B. 3
C. >3
D. Not considered
E. Some other options, please specify
3. You would consider a patient with advanced UC fit for carboplatin if the neuropathy is
A. ≤Grade 2
B. Grade 2
C. Grade 3
D. ≥Grade 3
E. Not considered
F. Some other options, please specify
4. You would consider a patient with advanced UC fit for carboplatin if the hearing loss is
A. ≤Grade 2
B. Grade 2
C. ≥Grade 2
D. ≥Grade 3
E. Not considered
F. Some other options, please specify
5. You would consider a patient with advanced UC fit for carboplatin if the cardiac status is
i. NYHA Heart Failure
A. >Class III
B.
C. Class III–IV
D. >Class IV
E. Not considered
F. Some other options, please specify
ii. Cardiac Ejection Fraction
A. <40%
B. 40%–50%
C. ≥50%
D. Not considered
E. Some other options, please specify
6. You would consider a patient with advanced UC fit for carboplatin if the hematological criteria are
A. Hemoglobin ≥11.0 g/dL
B. Platelet count ≥100,000´10^9^/L or 100,000/mm^3^
C. Neutrophil count ≥1500´10^9^/L or 1500/mm^3^
D. All of the above
E. Not considered
F. Some other options, please specify

Data collection and analysis

The online survey was structured with all mandatory fields, which rejected counting the partial and incomplete responses. Only complete responses were registered and subsequently considered for further calculations. The data collected from the responses were collated and descriptively analyzed. The responses entered by the participants in Microsoft Office Forms were automatically processed by Google Sheets, generating numbers, and graphs. The criteria for a parameter were chosen based on the option that received the highest response and was represented as count (n) and percentage (%), respectively.

## Results

Participants’ demographics

**Figure 1:**
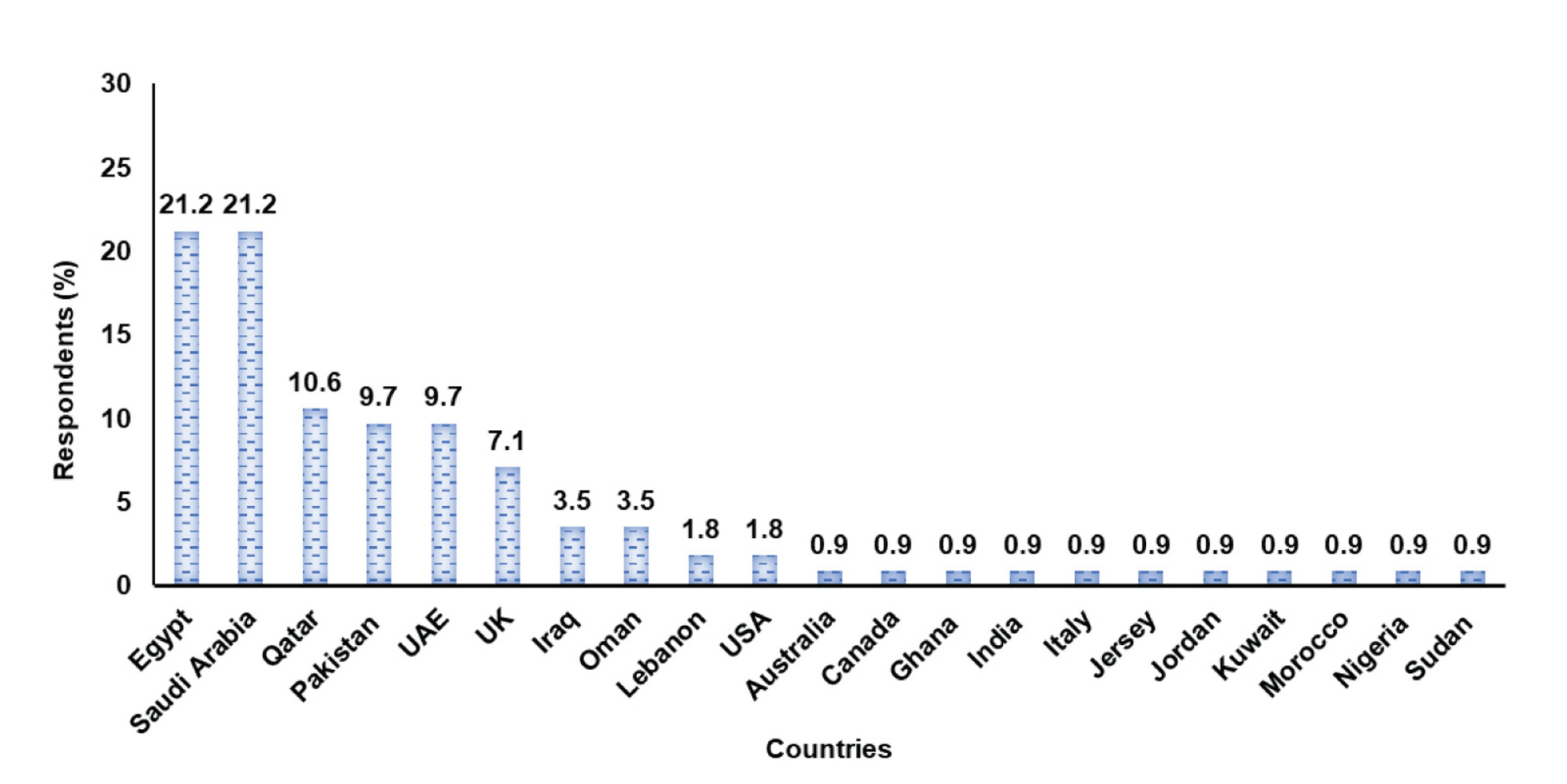
Country of practice of the respondents

**Figure 2:**
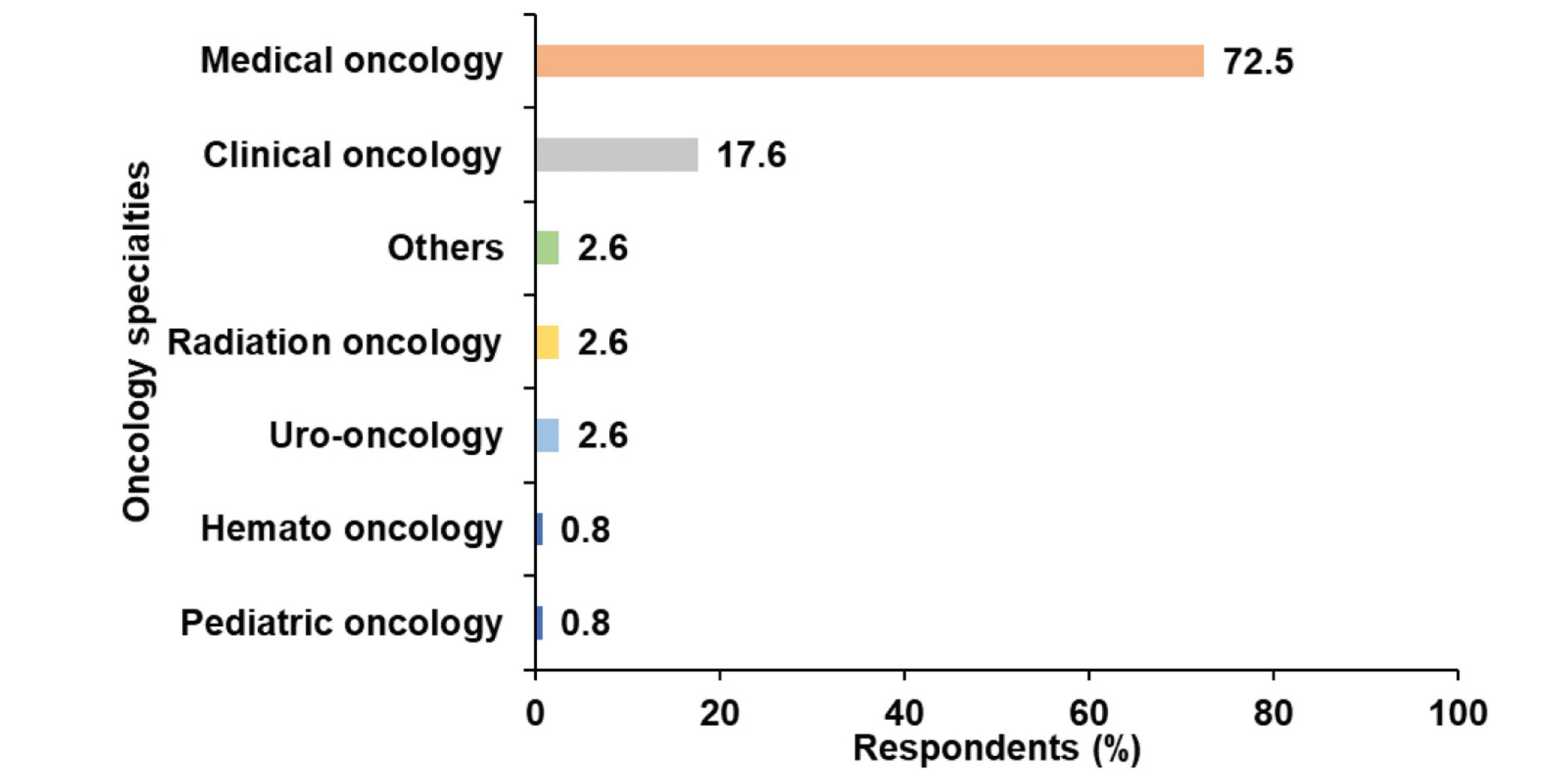
Specialization of the respondents

The survey garnered 136 responses from 21 countries, with 83% (n=113) being oncologists treating patients with mUC and 17% (n=23) not involved in mUC treatment (Table 2). Of the 113 respondents, the majority were from Egypt, Saudi Arabia, Qatar, Pakistan, and the UAE (Figure 1). Approximately 72.5% worked in medical oncology, followed by 17.6% in clinical oncology, while the remaining respondents represented various oncology specialties (Figure 2).

**Table 2:** Demographic characteristics of the respondents mUC: Metastatic Urothelial Carcinoma

Demographics	Respondents n (%)
Total responses	136
Treating patients with mUC	113 (83)
Not involved in mUC treatment	23 (17)
Gender	
Male	90 (79.6)
Female	23 (20.3)
Hospital associated	
Private	18 (15.9)
Public	57 (50.4)
University	38 (33.6)
Years of experience	
1-10	37 (32.7)
10-20	50 (44.2)
20-30	16 (14.1)
30-40	7 (6.1)
Number of patients treated annually	
<10	36 (31.8)
10-30	48 (42.4)
<30	29 (25.6)
Preferred first-line treatment	
Full-dose cisplatin	91 (80.5)
Split-dose cisplatin	23 (20.3)
Carboplatin	14 (12.3)
Others	5 (4.4)

Among the respondents, 79.6% were male and 20.3% were female. Most of them were associated with public hospitals (50.4%), followed by university (33.6%) and private (15.9%) hospitals. Participants had diverse experience levels, with 44.2% having 10-20 years of expertise in mUC treatment. Approximately 42.4% treated 10-30 patients annually, and 25.6% over 30 patients. The preferred first-line treatment was typically full-dose cisplatin (80.5%), followed by split-dose cisplatin (20.3%), carboplatin (12.3%), and other treatments (4.4%) (Table 2).

Participants’ responses

Respondents considered several critical criteria in assessing the eligibility of patients with mUC for full-dose cisplatin. Most (85.8%) were willing to assess a patient’s eligibility for full-dose cisplatin with creatinine clearance ≥60 mL/min, while 5.3% agreed to ≥40 mL/min. Respondents prioritized the overall health of patients, with 73.4% favoring ECOG PS ≤1 and 20.3% favoring ≤2. For neurotoxicity, 69.9% preferred ≤Grade 1 neuropathy and 25.6% ≤Grade 2. Hearing loss at ≤Grade 1 was emphasized by 67.2%, followed by 19.4% at ≤Grade 2. Cardiac status was a key concern, as 67.2% preferred a New York Heart Association (NYHA) heart failure ≤Class II, followed by 12.3% in ≤Class III, with 82.3% prioritizing a cardiac ejection fraction of ≥50%, and 10.6% considering 40-50% (Table 3). Hematological parameters were consistently underscored, with most respondents considering normal blood parameters such as hemoglobin levels ≥11.0 g/dL (69.9%), platelet counts ≥100,000/mm^3^ (89.3%), and neutrophil counts ≥1500/mm^3^ (93.8%) as crucial (Table 4).

**Table 3:** Criteria for considering full-dose cisplatin in patients with mUC ECOG PS: Eastern Cooperative Oncology Group Performance Status; mUC: Metastatic Urothelial Carcinoma; NYHA: New York Heart Association

Full-Dose Cisplatin	Respondents n (%)
Creatinine Clearance	
≥60 mL/min	97 (85.8)
≥30 mL/min	2 (1.7)
≥40 mL/min	8 (5.3)
Not considered	3 (2.6)
Other	5 (4.4)
ECOG PS	
0	5 (4.4)
≤1	83 (73.4)
≤2	23 (20.3)
≤3	1 (0.8)
Not considered	1 (0.8)
Neuropathy	
≤Grade 1	79 (69.9)
≤Grade 2	29 (25.6)
Not considered	5 (4.4)
Hearing Loss	
≤Grade 1	76 (67.2)
≤Grade 2	22 (19.4)
Not considered	15 (13.2)
NYHA Heart Failure	
≤Class II	76 (67.2)
≤Class III	14 (12.3)
Not considered	22 (19.4)
None	1 (0.8)
Cardiac Ejection Fraction	
<40%	2 (1.7)
40%-50%	12 (10.6)
≥50%	93 (82.3)
Not considered	6 (5.3)

**Table 4:** Hematological criteria for considering full-dose cisplatin in patients with mUC mUC: Metastatic Urothelial Carcinoma

Hematological Criteria	Considered n (%)	Not Considered n (%)
Hemoglobin ≥11.0 g/dL	79 (69.9)	34 (30.0)
Platelet count ≥100,000 x 10^9^/L or 100,000/mm^3^	101 (89.3)	12 (10.6)
Neutrophil count ≥1500 x 10^9^/L or 1500/mm^3^	106 (93.8)	7 (6.1)

For split-dose cisplatin eligibility, 52.2% selected creatinine clearance ≥40 mL/min, while 30.9% emphasized ≥60 mL/min. Evaluating ECOG PS, 56.6% preferred ≤2, followed by 32.7% considering ≤1. Neurotoxicity preferences were 49.5% ≤Grade 1, followed by 36.2% ≤Grade 2. For hearing loss, 54.8% considered ≤Grade 1, and 24.7% ≤Grade 2. Cardiac status preferences were NYHA heart failure ≤Class II (61.0%), followed by 15% considering ≤Class III. Regarding cardiac ejection fraction, 46.9% selected ≥50%, and 37.1% consented to 40-50% (Table 5). Hematological criteria analysis indicated that 54.8% adhered to normal hemoglobin ≥11.0 g/dL, and 77.8% and 79.6% considered normal platelet and neutrophil counts in patient selection, respectively (Table 6).

**Table 5:** Criteria for considering split-dose cisplatin in patients with mUC ECOG PS: Eastern Cooperative Oncology Group Performance Status; mUC: Metastatic Urothelial Carcinoma; NYHA: New York Heart Association

Split-dose Cisplatin	Respondents n (%)
Creatinine Clearance	
≥30 mL/min	6 (5.3)
≥40 mL/min	59 (52.2)
≥50 mL/min	1 (0.8)
≥60 mL/min	35 (30.9)
50-60 ml/min	1 (0.8)
Not considered	9 (7.9)
Other	2 (1.7)
ECOG PS	
≤1	37 (32.7)
≤2	64 (56.6)
≤3	3 (2.6)
0-1	1 (0.8)
Not considered	7 (6.1)
Others	1 (0.8)
Neuropathy	
≤Grade 1	56 (49.5)
≤Grade 2	41 (36.2)
≤Grade 3	4 (3.5)
Not considered	11 (9.7)
Others	1 (0.8)
Hearing Loss	
≤Grade 1	62 (54.8)
≤Grade 2	28 (24.7)
≤Grade 3	5 (4.4)
Not considered	17 (15.0)
Others	1 (0.8)
NYHA Heart Failure	
≤Class II	69 (61.0)
≤Class III	17 (15.0)
≤Class IV	1 (0.8)
Not considered	24 (21.2)
Others	2 (1.7)
Cardiac Ejection Fraction	
<40%	3 (2.6)
40%-50%	42 (37.1)
≥50%	53 (46.9)
Not considered	14 (12.3)
Others	1 (0.8)

**Table 6:** Hematological criteria for considering split-dose cisplatin in patients with mUC mUC: Metastatic Urothelial Carcinoma

Hematological Criteria	Considered n (%)	Not Considered n (%)	No Response n (%)
Hemoglobin ≥11.0 g/dL	62 (54.8)	50 (44.2)	1 (0.8)
Platelet count ≥100,000 x10^9^/L or 100,000/mm^3^	88 (77.8)	23 (20.3)	2 (1.7)
Neutrophil count ≥1500x 10^9^/L or 1500/mm^3^	90 (79.6)	21 (18.5)	2 (1.7)

For considering carboplatin as first-line treatment for mUC, 40.7% specified a creatinine clearance ≥30 mL/min, followed by 30.9% accepting ≥40 mL/min. Approximately, 70.7% emphasized ECOG PS ≤2, followed by 17.6% considering ≤1. Neurotoxicity preferences were 55.7% for ≤Grade 2, followed by 17.6% for ≤Grade 1 and 14.1% considering ≤Grade 3 neuropathy. Approximately, 46.9% accepted hearing loss ≤Grade 2, followed by 19.4% preferring ≤Grade 1 and 18.5% considering ≤Grade 3. Regarding NYHA heart failure, 43.3% favored ≤Class II followed by 34.5% considering ≤Class III along with 48.6% prioritizing a cardiac ejection fraction ≥50%, and 18.5% considering 40-50% (Table 7). Hematological criteria analysis revealed that 62.8%, 84.9%, and 91.1% considered normal hemoglobin, platelet, and neutrophil count criteria as important for the selection of patients (Table 8).

**Table 7:** Criteria for considering carboplatin in patients with mUC ECOG PS: Eastern Cooperative Oncology Group Performance Status; mUC: Metastatic Urothelial Carcinoma; NYHA: New York Heart Association

Carboplatin	Respondents n (%)
Creatinine Clearance	
≥20 mL/min	23 (20.3)
≥30 mL/min	46 (40.7)
≥40 mL/min	35 (30.9)
≥60 mL/min	3 (2.6)
Not considered	4 (3.5)
Other	2 (1.7)
ECOG PS	
0	1 (0.8)
≤1	20 (17.6)
≤2	80 (70.7)
≤3	10 (8.8)
Not considered	1 (0.8)
Others	1 (0.8)
Neuropathy	
≤Grade 1	20 (17.6)
≤Grade 2	63 (55.7)
≤Grade 3	16 (14.1)
Not considered	14 (12.3)
Hearing Loss	
≤Grade 1	22 (19.4)
≤Grade 2	53 (46.9)
≤Grade 3	21 (18.5)
Not considered	17 (15)
NYHA Heart Failure	
Class I	1 (0.8)
≤Class II	49 (43.3)
≤Class III	39 (34.5)
≤Class IV	6 (5.3)
Not considered	18 (15.9)
Cardiac Ejection Fraction	
<40%	14 (12.3)
40%-50%	21 (18.5)
≥50%	55 (48.6)
Not considered	22 (19.4)
Others	1 (0.8)

**Table 8:** Hematological criteria for considering carboplatin in patients with mUC mUC: Metastatic Urothelial Carcinoma

Hematological Criteria	Considered n (%)	Not Considered n (%)	No Response n (%)
Hemoglobin ≥11.0 g/dL	71 (62.8)	40 (35.3)	2 (1.7)
Platelet count ≥100,000 x 10^9^/L or 100,000/mm^3^	96 (84.9)	16 (14.1)	1 (0.8)
Neutrophil count ≥1500 x 10^9^/L or 1500/mm^3^	103 (91.1)	9 (7.9)	1 (0.8)

## Discussion

Bladder cancer imposes a significant burden on the healthcare system owing to its high prevalence and a 31-78% five-year recurrence rate after initial treatment [15]. While men are 3-4 times more prone to developing bladder cancer, women experience a much worse prognosis [1]. The five-year relative survival rate for individuals diagnosed with stage IV bladder cancer remains low, at approximately 15% [1]. The Galsky and Bajorin risk factor criteria guide decision-making for treatment options in mUC, particularly deeming patients ineligible for platinum-based chemotherapy [13].

In determining platinum-based chemotherapy eligibility for patients with mUC, respondents in our survey consistently prioritized specific criteria for each chemotherapy regimen. For first-line treatment with full-dose cisplatin, the predominant preferences included a creatinine clearance of ≥60 mL/min (85.8%), ECOG PS ≤1 (73.4%), neuropathy ≤Grade 1 (69.9%), hearing loss ≤Grade 1 (67.2%), NYHA heart failure ≤Class II (67.2%), and cardiac ejection fraction ≥50% (82.3%). Normal hematological parameters such as hemoglobin ≥11.0 g/dL (69.9%), platelet count ≥100,000/mm^3^ (89.3%), and neutrophil count ≥1500/mm^3^ (93.8%) were also emphasized (Table 9). For split-dose cisplatin eligibility, respondents prioritized criteria such as creatinine clearance ≥40 mL/min (52.2%), ECOG PS ≤2 (56.6%), neuropathy ≤Grade 1 (49.5%), hearing loss ≤Grade 1 (54.8%), NYHA heart failure ≤Class II (61.0%), and cardiac ejection fraction ≥50% (46.9%). Normal blood parameters were also considered essential such as hemoglobin ≥11.0 g/dL (54.8%), platelet count ≥100,000/mm^3^ (77.8%), and neutrophil count ≥1500/mm^3^ (79.6%) (Table 9). To determine the eligibility for carboplatin, the respondents’ favored criteria were creatinine clearance ≥30 mL/min (40.7%), ECOG PS ≤2 (70.7%), neuropathy ≤Grade 2 (55.7%), hearing loss ≤Grade 2 (46.9%), NYHA heart failure ≤Class II (43.3%), and cardiac ejection fraction ≥50% (48.6%). Hematological parameters were considered crucial such as hemoglobin ≥11.0 g/dL (62.8%), platelet count ≥100,000/mm^3^ (84.9%), and neutrophil count ≥1500/mm^3^ (91.1%) (Table 9).

**Table 9:** Preferred platinum eligibility criteria for patients with mUC Chosen criteria: This refers to the option that received the maximum preference from the respondents in the survey; ECOG PS: Eastern Cooperative Oncology Group Performance Status; mUC: Metastatic Urothelial Carcinoma; NYHA: New York Heart Association

Parameters	Full-Dose Cisplatin Chosen Criteria n (%)	Split-Dose Cisplatin Chosen Criteria n (%)	Carboplatin Chosen Criteria n (%)
Creatinine clearance (mL/min)	≥60 97 (85.8)	≥40 59 (52.2)	≥30 46 (40.7)
ECOG PS	≤1 83 (73.4)	≤2 64 (56.6)	≤2 80 (70.7)
Neuropathy	≤Grade 1 79 (69.9)	≤Grade 2 56 (49.5)	≤Grade 2 63 (55.7)
Hearing loss	≤Grade 1 76 (67.2)	≤Grade 1 62 (54.8)	≤Grade 2 53 (46.9)
NYHA Heart Failure	≤Class II 76 (67.2)	≤Class II 69 (61.0)	≤Class II 49 (43.3)
Cardiac ejection fraction (%)	≥50 93 (82.3)	≥50 53 (46.9)	40-50 55 (48.6)
Hemoglobin ≥11.0 g/dL	79 (69.9)	62 (54.8)	71 (62.8)
Platelet count ≥100,000 x 10^9^/L	101 (89.3)	88 (77.8)	96 (84.9)
Neutrophil count ≥1500 x 10^9^/L	106 (93.8)	90 (79.6)	103 (91.1)

Another multi-institutional study compared the impact of split-dose cisplatin on pathologic response rates in neoadjuvant chemotherapy for bladder cancer. Although the split-dose cisplatin group showed lower complete response rates, this difference was not statistically significant. Comparable response rates and adverse events, particularly nephrotoxicity, suggested careful patient selection for this dosing regimen [16]. In yet another study, the impact of the glomerular filtration rate on full-dose cisplatin-based neoadjuvant chemotherapy outcomes was investigated. Patients with normal glomerular filtration rate (≥60 mL/min) demonstrated a higher complete pathologic response rate (24% vs. 14%) than those with a lower glomerular filtration rate (34-59 mL/min). No significant glomerular filtration rate change was found after neoadjuvant chemotherapy [17]. A 2022 survey with 60 oncologists identified ECOG PS and renal impairment as crucial factors in platinum ineligibility for mUC [14]. The proposed updated criteria recommend ECOG PS >3, creatinine clearance <30 mL/min, peripheral neuropathy ≥Grade 2, and NYHA heart failure >Class III for platinum ineligibility, although age was not considered a significant criterion [14]. Additionally, carboplatin was preferred for renal failure owing to its reduced nephrotoxicity. However, the lack of scientific evidence underscores the need to establish criteria for a carboplatin treatment regimen.

Evidence suggests that carboplatin has significantly lower nephrotoxicity and neurotoxicity than cisplatin. Studies comparing cisplatin and carboplatin chemotherapy regimens for mUC provide insights into the platinum eligibility criteria. A phase II study comparing carboplatin and cisplatin regimens for metastatic bladder cancer found that carboplatin has lower nephrotoxicity and neurotoxicity [18]. Carboplatin showed an improved overall response rate with comparable progression-free survival and median overall survival. The thrombocytopenia was mild and reversible. Carboplatin was better tolerated with no severe nephrotoxicity discontinuations [18]. In another comparative study, carboplatin and cisplatin showed no significant differences in overall response rate or complete responses [19]. Despite the carboplatin group having more patients with advanced cancer experiencing bone metastasis, no renal toxicity was evidenced as compared to the cisplatin group. Furthermore, both groups had hematologic toxicities, but carboplatin-associated toxicities were more tolerable. Carboplatin-treated patients demonstrated better tolerance with similar outcomes to cisplatin-treated patients [19]. In one study, carboplatin-treated patients showed better tolerance, fewer severe toxicities, similar overall response rate, overall survival, and progression-free survival outcomes than cisplatin-treated patients [20]. Another study considering patients with adequate renal function and ECOG PS 0-2 found similar efficacy in advanced urothelial carcinomas, but carboplatin had fewer nephrotoxicity-related adverse events, suggesting that it may be a viable alternative, especially in cases of moderate renal failure [21].

Carboplatin is positioned as a less nephrotoxic substitute for renal-compromised patients than carboplatin [22-24]. Carboplatin’s pharmacokinetic profiles exhibit linear kinetics in individuals with creatinine clearances ≥60 mL/min [25,26]. The reported total body clearance, apparent volume of distribution, and mean residence time were 73 mL/min, 16 L, and 3.5 hours, respectively [26,27]. These pharmacological attributes contribute to the interest in carboplatin for its diminished nephrotoxicity and its predictable and consistent pharmacokinetic behavior, especially in patients with compromised renal function [25,27,28].

In our survey, the respondents preferred specific criteria based on the chemotherapy regimen for platinum eligibility. For full-dose cisplatin, they prioritized factors such as renal function, performance status, neuropathy, hearing levels, heart failure classification, and cardiac ejection fraction. Additionally, they emphasized the significance of normal hematological parameters, including hemoglobin, platelet count, and neutrophil count to be crucial.

Strengths and limitations

Despite providing valuable insights, this study has some limitations. The approach of self-reported data in survey-based studies may introduce bias, as respondents may provide information influenced by subjective interpretation. Additionally, despite extensive research on new oncology drugs in the United States, Europe, Canada, and Australia, responses from oncologists from these countries were limited (<20). The responses collated in this survey were predominantly from Asian oncologists. This is the main limitation of the survey in that it reflects the practice recommendations from a small number of oncologists, predominantly from Asia. This could affect the accuracy and reliability of the results. Further research integrating clinical outcomes and standardizing the criteria is essential for a comprehensive understanding of platinum-based chemotherapy in mUC.

## Conclusions

This study highlights the importance of specific treatment criteria in managing bladder cancer, especially mUC. Oncologists prioritize parameters for each chemotherapy regimen, emphasizing renal function, performance status, and hematological factors for platinum eligibility. Hematological parameters play a crucial role in patient assessment. The findings contribute to the evolving mUC treatment landscape, emphasizing the need for tailored approaches aligned with global oncologists’ perspectives. While carboplatin and cisplatin are established for front-line mUC treatment, studies support carboplatin as a less toxic alternative. Standardized criteria and larger samples are necessary for a more comprehensive understanding.
